# Skin Wound-Healing Potential of Polysaccharides from Medicinal Mushroom *Auricularia auricula-judae* (Bull.)

**DOI:** 10.3390/jof7040247

**Published:** 2021-03-25

**Authors:** Sariya Mapoung, Sonthaya Umsumarng, Warathit Semmarath, Punnida Arjsri, Pilaiporn Thippraphan, Supachai Yodkeeree, Pornngarm Limtrakul (Dejkriengkraikul)

**Affiliations:** 1 Department of Biochemistry, Faculty of Medicine, Chiang Mai University, Chiang Mai 50200, Thailand; srmapoung@gmail.com (S.M.); warathit_semmarath@cmu.ac.th (W.S.); punnida_dream@hotmail.com (P.A.); tipprapant@gmail.com (P.T.); yodkeelee@hotmail.com (S.Y.); 2 Center for Research and Development of Natural Products for Health, Chiang Mai University, Chiang Mai 50200, Thailand; sonthaya.u@cmu.ac.th; 3 Division of Veterinary Preclinical Sciences, Department of Veterinary Biosciences and Veterinary Public Health, Faculty of Veterinary Medicine, Chiang Mai University, Chiang Mai 51000, Thailand

**Keywords:** medicinal mushroom, *Auricularia auricula-judae*, wound healing, polysaccharide-rich extract, keratinocytes proliferation, fibroblasts proliferation, collagen synthesis, in vivo skin wound healing

## Abstract

*Auricularia auricula-judae*, a nutrient-rich mushroom used in traditional medicine, is a macrofungi that exhibits various biological properties. In this study, we have reported on the mechanisms that promote the wound-healing effects of a water-soluble polysaccharide-rich extract obtained from *A. auricula-judae* (AAP). AAP contained high amounts of polysaccharides (349.83 ± 5.00 mg/g extract) with a molecular weight of 158 kDa. The main sugar composition of AAP includes mannose, galactose, and glucose. AAP displayed antioxidant activity in vitro and was able to abort UVB-induced intracellular ROS production in human fibroblasts in cellulo. AAP significantly promoted both fibroblast and keratinocyte proliferation, migration, and invasion, along with augmentation of the wound-healing process by increasing collagen synthesis and decreasing E-cadherin expression (All *p* < 0.05). Specifically, the AAP significantly accelerated the wound closure in a mice skin wound-healing model on day 9 (2.5%AAP, *p =* 0.031 vs. control) and day 12 (1% and 2.5%AAP with *p =* 0.009 and *p* < 0.001 vs. control, respectively). Overall, our results indicate that the wound-healing activities of AAP can be applied in an AAP-based product for wound management.

## 1. Introduction

The wound-healing process is a complex sequence of cellular and molecular processes that consist of inflammation, new tissue formation, and tissue remodeling [1,2]. In brief, there are four stages of the wound-healing process. Hemostasis is the first immediate step that occurs at the wound site and requires platelets to repair damaged blood vessels through the process of blood clotting. The second step involves the inflammation utilized by neutrophils and macrophages in response to tissue injury via cytokine release and phagocytosis [3]. On approximately the 4th day after wounding, the proliferation phase begins. This process is characterized by the involvement of the cells embedded in the skin layers including specialized fibroblasts, which secrete a collagen framework onto the dermal skin layer in order to facilitate dermal regeneration. In addition, at the epidermal layer, keratinocytes are the cells responsible for epithelialization of the stratum corneum at skin layer resulting in the epidermal regeneration and the wound contraction. Lastly, the remodeling step involves the remodeling and realignment of the collagen tissue. This step is essential in establishing the greater tensile strength of the skin [4,5].

Regarding the cellular mechanism of the wound-healing process, tremendous progress has been made in recent years in identifying the critical events responsible for wound healing. Acute and chronic wounds involve a variety of interactions with components of the dermis and epidermal regeneration. Fibroblast cells play an important role in facilitating the proliferative stage via the production of various enzymes that degrade the fibrin clot, then replace it with extracellular matrix (ECM) components (such as collagen and hyaluronic acid) and infiltrate the margins of the wound [4]. Not only fibroblasts that are essential for the wound-healing process, but keratinocytes are indeed involved in critical steps, such as the epidermal regeneration and the closure of the wound. These steps contribute to the restoration of skin functions and mechanistically required the proliferation, migration, and differentiation of keratinocytes at the margins of the wound [1,6]. Despite the fact that the regular wound-healing process can simultaneously occur, many of intrinsic and extrinsic factors can interfere with the wound-healing process that can then result in an ultimate delay of wound repair. These factors include infection, necrotic tissue, vascular supply, co-existing physical factors (nutritional status), and disease states (diabetes, cancer, and arthritis) [7]. Therefore, an attempt to identify any remedies that could effectively accelerate skin wound healing would be of significant interest. 

In recent years, several researchers have investigated various kinds of natural products with regard to their wound-healing properties. These studies were conducted by targeting relevant potential wound repair mechanisms such as the activation of epithelial cells- or fibroblasts proliferation and migration, the inhibition of reactive oxygen species (ROS) production, and the modulation of inflammatory mediators [8]. Interestingly, the wound-healing properties of plant and fungi extracts have been reported by several authors. For instance, in plants, the aqueous leaf extract of *Ficus exasperata* (Moraceae) demonstrated wound-healing activity in rats with chronic diseases [9]. The perennial plant, *Cissus quadrangularis*, displayed wound-healing properties in mice with the skin structure of the wound area being completely returned to physiological state [10]. Moreover, in mushrooms, *Antrodia camphorata* rich in total polyphenols and flavonoids, significantly promoted wound healing in vitro by promoting fibroblast cell proliferation and increasing collagen synthesis in injured tissues in vivo with less inflammatory cell accumulation [11]. Moreover, the topical application of 10% *w*/*w* polysaccharide-rich extract derived from the Lingzhi mushroom, *Ganoderma lucidumm*, could enhance wound healing in STZ-induced diabetic rats [12]. Despite the increasing number of studies on the wound-healing activity of mushrooms, the wound-healing mechanism has not yet been fully identified.

*Auricularia auricula-judae* is a species of edible Auriculariales mushroom. It belongs to the family Auriculariaceae and can be identified by its ear-like shape and brown color. It is commonly referred to as Jew’s ear or (black) wood ear mushroom. It is widely distributed throughout Europe, East-, South-, and Southeast Asia. Besides possessing proteins and minerals, *Auricularia auricula* largely contains carbohydrates. The recent reports have shown that *A. auricula* extract exhibited antioxidant activity and promoted procollagen biosynthesis [13], antitumor activities [14], and anticoagulant activity [15]. However, the medicinal properties of *A. auricula-judae* as a wound-healing agent has not yet been fully elucidated, as only a few studies have been conducted so far. Therefore, this study aimed to investigate the wound-healing potential and underlying mechanism of the polysaccharide-rich extract of *A. auricula-judae* in both in vitro and in vivo experiments.

## 2. Materials and Methods

### 2.1. Reagents and Chemicals

Dulbecco’s modified Eagle medium (DMEM), trypsin, and penicillin-streptomycin were supplied by Gibco (Grand Island, NY, USA). Fetal bovine serum (FBS), RIPA buffer, protease inhibitors, and Coomassie Plus™ Protein Assay Reagent were obtained from Thermo Scientific Company (Waltham, MA, USA). Nitrocellulose membrane and Enhanced chemiluminescent (ECL) reagent were supplied by GE Healthcare (Little Chalfont, UK). Antibody specific to E-cadherin was purchased from Santa Cruz Biotechnology (Santa Cruz, CA, USA). Furthermore, 3-(4,5-dimethylthiazol-2-yl)-2,5-diphenyltetrazolium bromide (MTT) dye, ABTS [2,2′-azino-bis (ethylbenzthiazoline-6-sulfonic acid)], 2′,7′-dichlorofluorescin diacetate (DCF-DA), fibronectin, and the antibody to β-actin were purchased from Sigma-Aldrich (St. Louis, MO, USA (Darmstadt, Germany). Sephacryl^TM^ S-400 high resolution was purchased from GE Healthcare Bio-Sciences AB (Uppsala, Sweden). Dextran blue derived from *Leuconostoc spp.* was purchased from Fluka Biochemika (Buchs, Switzerland). Gel filtration standard components (Thyroglobulin, γ-globulin, Ovalbulmin, Myoglobulin, and Vitamin B12) were purchased from Bio-Rad Laboratories, Inc. (Hercules, CA, USA).

### 2.2. Cell Culture

HaCat cell line (human keratinocytes) was purchased from ATCC (Virginia, United States). Primary human skin fibroblasts were aseptically isolated from an abdominal scar after a surgical procedure involving a Caesarean section that had been performed at the surgical ward of Chiang Mai Maharaj Hospital, Chiang Mai University, Thailand (Study code: BIO-2558-03549 approved of by Medical Research Ethics Committee, Chiang Mai University). The methods of primary fibroblast preparation and culturing were employed following the previously described protocol [16]. All cell types were cultured in DMEM with 10% FBS, and supplemented with 100 U/mL penicillin, and 100 µg/mL streptomycin. The cells were maintained in a 5% CO_2_ humidified incubator at 37 °C.

### 2.3. Animals

Six- to eight-week-old BALB/c mice (male, weight = 28–30 g) were purchased from Nomura Siam International, Bangkok, Thailand. Mice were acclimatized to the animal housing laboratory for at least 7 days. The animals were housed in specific pathogen-free facilities and handled according to the institutionally recommended guidelines. All animals were maintained under a constant 12 h/12 h light-dark cycle with humidity levels maintained at 60 ± 10% at 25 ± 1 °C. All mice were supplied with free access to water and food. 

### 2.4. A. auricula-judae Extraction

Fresh *A. auricula-judae* was harvested by Siri Mushroom Organic Farm in Thung Hua Chang District, Lumphun Province, Thailand. The voucher specimen number of *A. auricula-judae* (No. 023241) was certified at the herbarium of the Flora of Thailand, Chiang Mai University, Thailand. The *A. auricula-judae* extraction was prepared by following the previously reported procedure with some modifications [17]. Briefly, the fruiting bodies of *A. auricula-judae* were washed and then oven-dried at 50 °C and finally ground into a fine powder. A 100 g sample of *A. auricula-judae* dried powder was suspended in 1 L of methanol for 2 h at 76 °C. Thereafter, the suspension was filtered through Whatman No.2 filter paper to remove the methanol-soluble materials. The residue was then resuspended in 1 L of distilled water for 2 h at 97 °C. The clear supernatant was collected after centrifugation at 7800× *g* for 15 min at 4 °C. This extraction step was repeated three times and the obtained supernatants were pooled and concentrated in a rotary evaporator. The concentrated supernatant was filtrated to remove any water-insoluble materials. The crude polysaccharide was obtained by precipitation in ethanol for 24 h, at 4 °C. The resulting precipitate was kept after centrifugation at 7800× *g* for 15 min at 4 °C. After that, the crude polysaccharide was dried at room temperature, dissolved in distilled water, and dialyzed against running water for 24 h. The non-dialyzed portion was centrifuged to remove any insoluble materials, then lyophilized and named as an *A. auricula-judae* polysaccharide-rich extract (AAP).

### 2.5. Molecular Weight of Polysaccharide Derived from A. auricula-judae Determination by Sephacryl S-400 Gel Filtration

To determine the molecular weight of AAP, the extract (2.5 mg) was dissolved in 0.5 mL of 0.2 M ammonium bicarbonate buffer (pH 7.0) and applied onto a Sephacryl S-400 HR column (1.7 × 130 cm) equilibrated with 0.2 M ammonium bicarbonate buffer. The column was eluted with the same buffer at a flow rate of 25 mL/h and the aliquots were collected as 5 mL per tube. These fractions were assayed for hexose sugars and for standard proteins, as will be described in the next section (wavelength absorbance of 490 nm and 280 nm, respectively) [17]. The column was calibrated using Blue dextran (average MW = 2000 kDa, for Vo), Phenol red (MW = 354 Da, for Vt), and standard proteins with different molecular masses (Thyroglobulin MW = 670 kDa; γ-globulin MW = 158 kDa; Ovalbumin MW = 44 kDa; Myoglobin MW = 17 kDa; and Vitamin B12 MW = 1.3 kDa). The molecular weight of AAP was calculated using the log molecular weight (logMW) curve of standard proteins. 

### 2.6. Total Carbohydrate Assay

The total carbohydrate content in AAP was determined using a Total Carbohydrate Assay Kit (Sigma-Aldrich, USA) according to the manufacturer’s protocol. Briefly, various concentrations of AAP (30 µL) and sulfuric acid (150 µL) were added to each of the tubes. The solutions were then mixed by pipetting and the reaction was incubated for 15 min at 90 °C in the dark. A developer was added to each well and they were mixed using a shaker for 5 min at room temperature. Subsequently, the contents were mixed for 1 min before being measured at an absorbance of 490 nm. 

### 2.7. Quantification of the Constituent Monosaccharides A. auricula-judae Using GC–MS

An AAP sample was prepared by following the previously described protocol for GC-MS analysis [18]. Dried samples were methanolized with 3 N HCl in methanol for 2 h at 121 °C followed by re-N-acetylation and tri-methylsilylation. An analysis of the constituent monosaccharides was performed by following the previously reported protocol [19]. Briefly, helium was used as the carrier gas at a flow rate of 1 mL/min. The temperature program was set to increase from an initial 120 to 200 °C, at 8 °C/min and held for 10 min before being heated at 6 °C/min to 230 °C. It was then held for a further 20 min. Peaks were identified and estimated using myoinositol as the internal standard. The quantity of fractions was determined from the peak area using response factors. The inlet temperature was kept constant at 210 °C and the MS transfer line was set at 270 °C. Mass spectrophotometer (MS) acquisition parameters included scanning from *m/z* 50 to 550 in the electron impact (EI) mode for routine analysis.

### 2.8. Antioxidant Activity by ABTS Assay

The antioxidant activity of the AAP was determined using ABTS assay. The ABTS radical cation was prepared by mixing 7 mM ABTS stock solution with 2.45 mM potassium persulfate (K_2_S_2_O_8_) (1/1, *v*/*v*). The mixture was incubated in the dark for 12–16 h until the reaction was completed. The assay was conducted on 990 µL of ABTS solution and 10 µL of the AAP (0–400 μg/mL), and vitamin E was used as a positive control in this assay. After 6 min of incubation, the absorbance was recorded immediately at 734 nm using a spectrophotometer. The percent inhibition of ABTS activity was calculated using the following equation:

(1) $$Inhibition\;of\;ABTS\;=\;\frac{Abs_{Control}-Abs_{Sample}}{Abs_{Control}}\;\times \;100$$

### 2.9. Intracellular ROS Determination

Intracellular ROS in cellulo upon UVB irradiation was determined using DCF-DA as has been previously described [20]. Primary fibroblasts (8.0 × 10^3^ cells/well) were seeded in a 96-well plate for 24 h. After that, cells were exposed to UVB at the intensity of 15 mJ/cm^2^ using the ultraviolet cross linker (CL-1000, UPV Inc., Upland, CA, USA). Fibroblast cells were then treated with or without increasing the concentrations of AAP (0–100 µg/mL) or 25 µg/mL of vitamin C (positive control) dissolved in culture medium for the duration of 24 h. Fibroblasts were then washed with PBS, 10 µM of DCF-DA was then added and the cells were incubated for a further 30 min. The fluorescent intensity was measured at an excitation wavelength of 485 nm and an emission wavelength of 525 nm. Intracellular ROS was calculated and compared to the control of the UVB-irradiated fibroblast cells.

### 2.10. Cell Proliferation Assay

The effects of AAP on cell proliferation were determined by MTT and trypan blue staining assays. For the MTT assay, the cells (8 × 10^3^ cells/well) were treated with increasing concentrations of AAP (0–25 μg/mL) in culture medium or culture medium alone (vehicle control) for 24 and 48 h. Following the AAP treatment, the cells were incubated with 10 μL of 0.5 mg/mL MTT in PBS for 4 h. The culture supernatant was then removed, and the culture was re-suspended with 200 μL of DMSO to dissolve the MTT formazan crystals. The absorbance was measured at 540 and 630 nm using a UV-visible spectrophotometer. The assay was performed in triplicate at each concentration. 

With regard to the trypan blue cell staining method, the cells (1.5 × 10^5^ cells/well) were seeded in a 6-well plate and incubated for 24 h. Further, the cells were treated with different concentrations of AAP (0–25 µg/mL) in culture medium or culture medium alone (vehicle control) and incubated for 24 and 48 h. Finally, cells were trypsinized and suspended in the culture medium. The cells were stained with 0.4% trypan blue dye at the ratio of 1:1. Cell proliferation for each concentration was determined using trypan blue dye and the resulting values were compared with the control. 

### 2.11. Scratch Assay

Wound-healing activity was examined using the modified scratch assay as has been previously described [21]. The primary fibroblast (2 × 10^5^ cells/well) or HaCat cells (2 × 10^5^ cells/well) were seeded into each well of a 6-well plate and incubated with completed DMEM medium at 37 °C and 5% CO_2_ for 24 h. After incubation, the monolayer confluent cells were scrapped horizontally with a sterile yellow pipette tip and washed with PBS. The cells were incubated with various concentrations of AAP (0–25 µg/mL) by diluting with 0.5% FBS-DMEM or 0.5% FBS-DMEM without AAP (vehicle control). The migration rates were observed, photographed under phase-contrast microscopy, and analyzed using IMAGE J 1.410. The percentage of the closed area was measured and then compared with the control.

### 2.12. Transwell Migration and Invasion Assays

The effect of AAP on human fibroblast and keratinocyte cell migration, and invasion, was determined by transwell assay as has been previously described [22,23]. Polyvinylpyrrolidone-free polycarbonate filters (8 μM pore size) (BD Biosciences, Franklin Lakes, NJ, USA) were coated with 10 μg/mL fibronectin (migration assay) along with 15 μg of matrigel per filter (invasion assay). The primary fibroblasts (1 × 10^5^ cells/well) and HaCat cells (2 × 10^5^ cells/well) were treated with various concentrations of AAP (0–25 µg/mL) in 0.5% FBS-DMEM or 0.5% FBS-DMEM without AAP (vehicle control) and seeded into the upper chamber. The 10% FBS-DMEM was added into the lower chamber as a chemoattractant. The cells were incubated for the next 48 h for both invasion and migration assays. The invading or migrating cells were fixed with 95% ethanol for 5 min. They were then stained with 0.5% crystal violet in 20% methanol for 30 min. The invading cells were photographed under phase-contrast microscopy (Nikon Eclipse TS100, Nikon Instrument Inc., Melville, NY, USA). The percentages of the areas occupied with cells were then determined with IMAGE J 1.410. The percentage of cell invasion or migration was measured and compared with control.

### 2.13. Collagen Synthesis Assay

The primary fibroblast cells (1 × 10^5^ cells/well) were seeded into 24-well plates for 24 h and incubated at 37 °C in an atmosphere of 5% CO_2_. After incubation, the cells were pre-treated with the 0.5%-FBS-DMEM medium for 24 h. The medium was then removed, and the cells were incubated with or without AAP (0–25 µg/mL) or vitamin C; 25 µg/mL (a positive control), in 0.5%-FBD-DMEM (vehicle control) for 48 h. After that, the cultured medium was kept and the collagen was determined using Sirius Red Collagen Staining Kit (Chondrex Inc., Redmond, WA, USA) according to the manufacturer’s protocol. Briefly, the proteins in the culture supernatant were precipitated using 25% tricholoacetic acid (TCA) and washed with 5% TCA. Proteins were dissolved in 0.05 M acetic acid. The collagen was then stained with Sirius red for 20 min at RT and washed with washing buffer. The collagen pellets were dissolved in an extraction buffer and were then measured at 540 nm using a UV-visible spectrophotometer. The collagen in each sample was calculated from the collagen standard curve and interpreted as the % of control.

### 2.14. Western Blot Analysis

The primary fibroblasts (2.0 × 10^5^ cells/well) and the HaCat keratinocyte cells (4.5 × 10^5^ cells/well) were treated with increasing concentrations of AAP for 24 and 48 h. After incubation, the cells were collected and lysed using RIPA buffer. Protein concentration levels were determined using the Bradford method. The whole-cell lysates were then subjected to 10% SDS-PAGE. The proteins were transferred onto nitrocellulose membranes and the membranes were blocked with 5% non-fat dried milk protein in 0.5% TBS-tween. After that, the membranes were further incubated overnight with the desired primary antibody at 4 °C followed by incubation with horseradish peroxidase-conjugated secondary antibody. Bound antibodies were detected using the chemiluminescent detection system and then exposed to the X-ray film (GE Healthcare Ltd., Little Chalfont, UK). The bands were normalized to β-actin as a loading control. Band density levels were analyzed using IMAGE J 1.410 (National Institutes of Health, Rockville, MD, USA).

### 2.15. In Vivo Wound-Healing Activity by Mice Skin Wound-Healing Model

An assessment of the wound lesion area was done to investigate the wound-healing activity of AAP. This was examined in the mice skin wound-healing model using the modified protocol that has been previously described [24]. In brief, BABL/c mice were subjected to full skin thickness excision. Mice were anesthetized before circular wounds of 5 mm in diameter were created on the shaved dorsum of the mice using a disposable biopsy punch (Kai medical, Japan). Twenty-one wounded mice were randomly divided into three groups. Every group was treated with either sterilized 0.9% normal saline (vehicle control) or an appropriate dose of AAP (1.0% *w*/*v*, or 2.5% *w*/*v*). To monitor the wound closure, photographs of the mice were taken every three days and the wound areas were measured using a digital vernier calipers (BEC, Taiwan). The wound area of each mouse was calculated using the following formula:

Area of wound (A) = π × (r_major diameter_ × r_minor diameter_) (2)

Experimental mice were sacrificed on day 12 of the experiment. Wounded tissues were then harvested and fixed for histological analysis with 10% formalin buffer solution and embedded in paraffin. A section of 4 μm thickness was prepared and stained with either hematoxylin and eosin (H&E) or Masson’s trichrome. The stained sections were observed under a digital whole-slide scanner (Pannoramic MIDI II machine, 3DHISTECH Ltd., Budapest, Hungary) and photographed (10×). 

### 2.16. Statistical Analysis

All data are presented as mean ± standard deviation (S.D.) or standard error (S.E.) values. Statistical analysis was conducted with GraphPad Prism 6.0 using independent *t*-test or one-way ANOVA with Dunnett’s test. Statistical significance was determined at *p* < 0.05, *p* < 0.01, and *p* < 0.001. 

## 3. Results

### 3.1. Preparation and the Polysaccharide Characteristics of A. auricula-judae Extract

**Figure 1:**
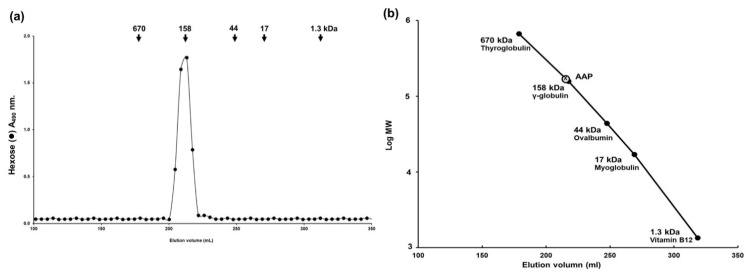
Polysaccharide characteristics of *A. auricula-judae* (AAP). The molecular weight of the polysaccharides was determined by Sephacryl S-400 gel filtration chromatography. (**a**) AAP was eluted with 0.2 M ammonium bicarbonate at a flow rate of 25 mL/h. The fractions were assayed for hexose sugars (A490 nm), and the standard proteins are represented by arrows to indicate molecular size (kDa). (**b**) The molecular weight was calculated using the calibration logMW curve of standard proteins (ranging from 1.3–670 kDa.).

AAP was prepared as has been described in Section 2.4. The% yield of AAP from *A. auricula-judae* mushroom was at 8.9% (*w*/*w*). The total amount of carbohydrates was 349.83 ± 5.00 mg/ g of AAP. AAP was not contaminated with phenolic nor flavonoid compounds as determined by Folin-Ciocalteu assay and aluminum chloride colorimetric assay, respectively (data not shown). The size of the polysaccharides obtained from AAP was determined for the molecular weight using Sephacryl S-400 gel filtration chromatography and the results are shown in Figure 1. The results indicate that the polysaccharides obtained from AAP displayed a single peak containing hexose sugar, which suggest the potential for polysaccharides to serve as a major compound of AAP (Figure 1a). AAP has a molecular weight at approximately 158 kDa as determined by the log molecular weight of the protein standard curve (Figure 1b). Additionally, the analyses of constituent monosaccharides using GC-MS technique indicated that those monosaccharides of AAP mainly consisted of mannose sugar (14.862% of total) followed by galactose (2.123%), and glucose (1.026%) with a molar ratio of 1.49:0.21:0.10, respectively.

### 3.2. AAP Displayed Antioxidant Activities In Vitro and in Cellulo

**Figure 2:**
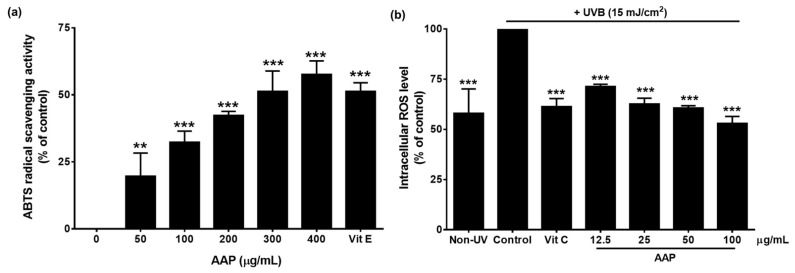
Antioxidant activities of AAP. The antioxidant activities of AAP were determined by ABTS assay (**a**) and DCF-DA fluorescent assay (**b**). For ABTS assay, the AAP (0–400 μg/mL), and Vit E at 15 μg/mL was used as a positive control. The intracellular ROS production was calculated as the percent of inhibition. For DCF-DA antioxidant activity, primary human skin fibroblasts were exposed to UVB at 15 mJ/cm^2^ using ultraviolet crosslinker. The intracellular ROS after UVB irradiation in fibroblasts was determined by DCF-DA dye. Vit C at 25 µg/mL was used as positive control. Data are represented as mean ± S.D. values of three independent experiments, ** *p* < 0.01 and *** *p* < 0.001 vs. the control.

The free radical-scavenging properties of AAP were determined by ABTS assay. As is shown in Figure 2a, AAP exhibited antioxidant activity in a dose-dependent manner with an effective concentration (EC_50_) of 226.67 ± 10.41 µg/mL, while the EC_50_ of vitamin E was at 15.48 ± 0.22 µg/mL. However, in the Figure 2a, the data are only shown for a dose of vitamin E as equal to the EC_50_ value. Additionally, the inhibitory effect of AAP on intracellular ROS production in UVB-irradiated primary fibroblasts was determined by DCF-DA fluorescent assay. The results showed that the intracellular ROS levels increased in the fibroblast cells by approximately 1.8-fold higher in control compared to non-UVB. As is shown in Figure 2b, vitamin C (25 µg/mL), which was used as a positive control, significantly decreased the intracellular ROS generation in UVB irradiated fibroblasts by approximately 1.6-fold when compared with the UVB-irradiated control group (*p* < 0.001). Moreover, AAP at 100 µg/mL significantly decreased the intracellular ROS generation in a dose-dependent manner by approximately two-fold (*p* < 0.001). 

### 3.3. AAP Induced Human Fibroblast and Keratinocyte Cell Proliferation

**Figure 3:**
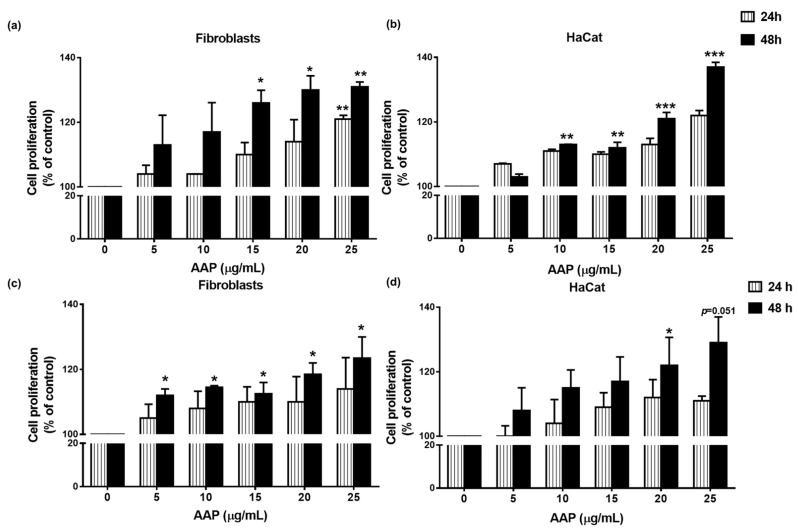
AAP induced fibroblast and keratinocyte cell proliferation. The effect of AAP on human fibroblasts and keratinocyte cell proliferation was determined by MTT assay (**a**,**b**) and trypan blue cell counting (**c**,**d**). The cells were treated with increasing concentrations of AAP for 24 and 48 h and cell proliferation was determined by MTT assay or trypan blue cell counting method. Data are represented as mean ± S.D. values of three independent experiments. * *p* < 0.05, ** *p* < 0.01, and *** *p* < 0.001 vs. control.

The effects of AAP (0–25 µg/mL) on human fibroblast and keratinocyte proliferation at 24 and 48 h were determined by MTT and trypan blue cell counting assays. Based on the DCF-DA antioxidant studies, it indicated the cell-based experiment and at 0–25 µg/mL of AAP provided significant decrease in intracellular ROS production. The MTT assay results are shown in Figure 3a,b. It was determined that the AAP treatment could significantly induce fibroblast (*p* < 0.01) and keratinocyte (*p* < 0.001) cell proliferation in a dose-dependent manner after 48 h. Similar to MTT, the trypan blue staining showed that AAP significantly induced human fibroblast cells proliferation in a dose-dependent manner (Figure 3c, *p* < 0.05). Whereas, the AAP dose dependently induce keratinocyte cell proliferation, but the statistical significance was only observed at the 20 µg/mL of AAP at 48 h (Figure 3d, *p* < 0.05 and *p =* 0.0511 for 25 µg/mL of AAP).

### 3.4. AAP Promotes Human Fibroblasts and Keratinocyte Cells Migration and Invasion Together with Augmentation of Collagen Synthesis and Decreasing E-Cadherin Expression

**Figure 4:**
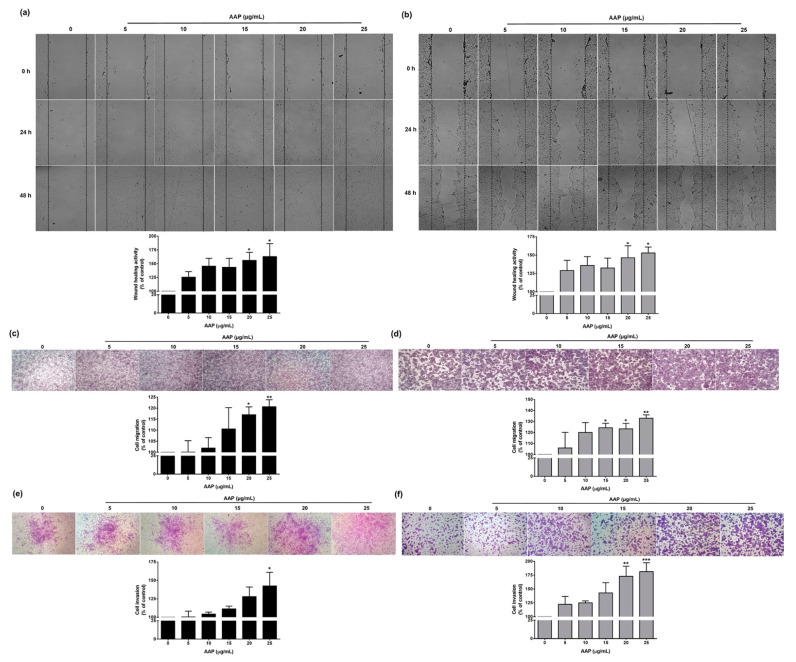
AAP promoted healing activity via facilitating migration and invasion in both human fibroblast and keratinocyte cells. The effects of AAP on human fibroblast and keratinocyte cells wound healing were determined by scratch assay ((**a**); fibroblasts and (**b**); HaCat cells and the wound-healing activity at 48 h was calculated as shown in the histogram inserts). Transwell assay was used to investigate human fibroblasts (**c**) and Hacat (**d**) cells migration (cell migration at 48 h was calculated as shown in the histogram inserts) and invasion ((**e**); fibroblast and (**f**); Hacat cells and cell invasion was calculated as shown in the histogram inserts). Data are presented as mean ± S.D. values of three independent experiments. * *p* < 0.05, ** *p* < 0.01, and *** *p* < 0.001 vs. control.

Promotion of the wound-healing effect is partially dependent upon fibroblast and keratinocyte activities through the migration and invasion processes. Thus, the induction of cell migration in the wound-healing activity was examined using scratch assay and transwell migration assay. As is shown in Figure 4a,b, each concentration of AAP at 5–25 µg/mL induced human fibroblast and keratinocyte cells migration in a dose-dependent manner and reaching statistical significance at higher doses (at 20 and 25 µg/mL for both cells, *p* < 0.05). Similar to the scratch assay, the transwell assay demonstrated that AAP at a concentration of 5–25 µg/mL increased fibroblast and keratinocyte cell migration in a dose-dependent manner and reaching statistical significance at higher doses (from 20 µg/mL in fibroblasts and from 15 µg/mL in keratinocytes, *p* < 0.05) as is shown in Figure 4c,d. Additionally, AAP could significantly induce fibroblast and keratinocyte cell invasion in a dose-dependent manner and reaching statistical significance at higher doses (at 25 µg/mL in fibroblasts and from 20 µg/mL in keratinocytes, *p* < 0.05), as is shown in Figure 4e,f. The above results could be summarized by stating that AAP induced fibroblast and keratinocyte cell invasion and migration, which could potentially lead to the promotion of wound-healing effects.

**Figure 5:**
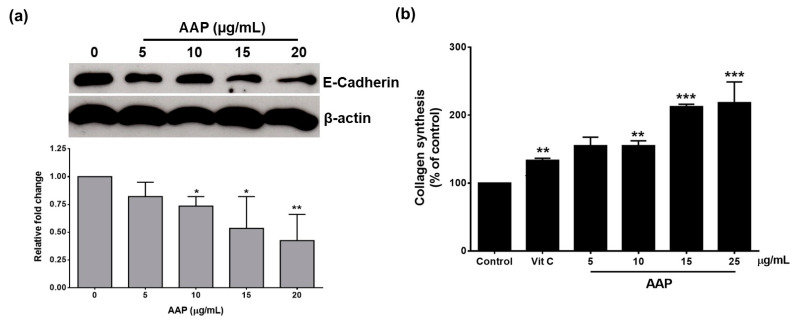
AAP decreased E-cadherin expression in keratinocytes and enhanced collagen synthesis in fibroblasts. The E-cadherin expression (**a**) in keratinocyte cells after AAP treatment for 24 h was determined by Western blot analysis. The bands were normalized to β-actin as a loading control. The quantification level of protein expression was determined by IMAGE J software. Collagen synthesis (**b**) was determined using a Sirius Red Collagen Detection kit. Human fibroblasts were starved in serum-free medium for 24 h. Cells were then treated with various concentrations of AAP (0–25 µg/mL) for 48 h. Data are presented as mean ± S.D. values of three independent experiments, ** *p* < 0.01 and *** *p* < 0.001 vs. control.

The effect of AAP on keratinocyte cell migration was confirmed by the expression of the E-cadherin, which is the protein biomarker of the epithelial cells and its downregulation could be used as a marker for epithelial cells migration/invasion. As is shown in Figure 5a, AAP decreased E-cadherin expression in keratinocytes. In addition to the decrease in the expression of epithelial biomarker in keratinocytes, the increase in collagen production in dermal fibroblasts was also determined in this study. The evaluation of collagen synthesis from human skin fibroblasts using a Sirius Red/Fast Green Collagen Staining Kit is shown in Figure 5b. Vitamin C, as a positive control, at a concentration of 25 µg/mL significantly increased collagen synthesis from human fibroblasts by approximately 40% when compared with the control (*p* < 0.01). As is shown in Figure 5b, AAP (0–25 μg/mL) could significantly increase collagen synthesis from fibroblasts in a dose-dependent manner (*p* < 0.01) with a compatible degree of activity when compared with vitamin C. Overall, AAP displayed a potent effect as a candidate molecule in wound-healing or wound-dressing agents.

### 3.5. AAP Accelerated Wound Closure in Mice Skin Wound-Healing Model

**Figure 6:**
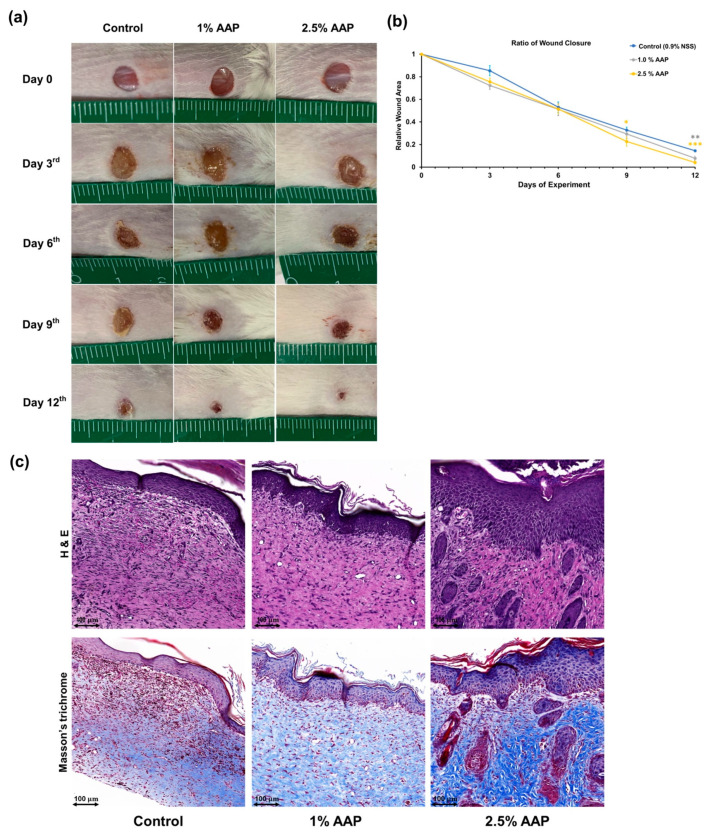
AAP accelerated wound closure and wound healing in vivo. (**a**) Photographs of the full thickness excision wounds in BALB/C mice in each group on day 0, 3, 6, 9, and 12 of the experiment. (**b**) The ratio of wound closure was expressed as relative wound area compared with that which was present on each day of the experiment. (**c**) H&E (**upper panel**) and Masson’s Trichrome (lower panel) stained wound tissues after 12 days of treatment with 0.9% sterilized normal saline solution (left), 1%*w*/*v* AAP (middle), and 2.5%*w*/*v* AAP (right). Data are expressed as Mean ± SE values, *n* = 7 for each group. *p < 0.05, ** *p* < 0.01, and *** *p* < 0.001 vs. vehicle control group.

To determine the wound-healing activities of AAP in vivo using a mice skin wound-healing model, the wound contraction rate was compared among the group of BALB/C mice receiving different treatments over the 12-day duration of the experiment (0.9% NSS, 1% or 2.5% (*w*/*v*) of AAP). The ratio of wound closure in each group along the period of the experiment was determined by monitoring the wound area as measured by digital vernier calipers. The wound contraction was traced on the 0, 3rd, 6th, 9th, and 12th days. The wound closure rate for each group was expressed as the ratio of wound area compared with that on day 0 (Relative wound area at day zero = 1), as is shown in Figure 6a,b. Our results indicate that, when compared with the vehicle control group, the wound closure in BALB/C mice receiving AAP resulted in significant rapid wound contractions on day 9 (only 2.5% AAP, *p* = 0.031) and day 12 (both 1% and 2.5% AAP with *p* = 0.009 and *p* < 0.001, respectively). The results of a histological study using H&E and Masson’s trichrome staining images in BALB/C mice after 12 days of AAP treatment are shown in Figure 6c. All mice wound skin sections in the control, and those in various treatment groups, did not reveal the signs of apoptosis, necrosis, or inflammatory cells infiltration indicating the validity of the mice skin wound-healing model. Furthermore, no toxicity of the AAP attested concentrations was observed. When compared with the vehicle control group, the AAP-treated groups showed the thickening of epidermis (in 2.5% AAP) and reduction of granulation tissue after both AAP treatment. The dermal layer of the H&E stained section showed the accumulation of fibroblast cells, and the Masson’s Trichrome stained section revealed the dense collagen network present after AAP treatment, especially at a concentration of 2.5%. Overall, AAP could accelerate the wound healing in vivo which has been demonstrated by a rapid wound closure rate. Furthermore, the wound architecture after AAP treatment revealed the thickening of skin’s epidermis together with the fibroblast accumulation and dense collagen network at the dermal skin layer.

## 4. Discussion

The results of our study indicate that AAP accelerates the wound-healing process through the promotion of fibroblast and keratinocyte proliferation, migration, and invasion, and the enhancement of collagen synthesis that occurs during the proliferation step of the wound repair process. As was consistent with our study, other medicinal mushroom and plant-based extracts have been verified for wound-healing properties with those targeting various mechanisms, including epithelial and dermal cells stimulation, reduction of reactive oxygen species (ROS), and modulation of inflammatory intermediates [25,26]. The efficiency of the wound-healing process is largely dependent upon the balance of proinflammatory and pro-regenerative signals that are mediated by cytokines [27,28]. These statements support our findings as AAP was found to display strong antioxidant properties that may have beneficial effects on balancing the pro- and anti- inflammatory mediators of the local wound environment. In previous studies, *A. auricula* mushroom extracts exhibited a variety of pharmacological properties such as antioxidant, blood lipid-lowering, anti-inflammation, antitumor, and anti-radiation activities [13,19,29,30,31]; however, there is no direct evidence for their wound-healing effects. The levels of ROS in tissue injuries are considered to be associated with the wound-healing processes. Low levels of ROS are essential for the stimulation of wound healing. However, high levels of ROS can impair ROS detoxification resulting in cellular damage and aggravation of the wound [32]. Moreover, intracellular ROS generation is one of the important factors that promote prolonged inflammation occurring in the surrounding wound margins [33]. Previously, experimental and clinical studies have indicated that those antioxidant and anti-inflammatory properties are considered beneficial elements at the initial stage of wound healing [32,34]. In this study, the antioxidant properties of AAP were confirmed by both in vitro ABTS assay and in cellulo DCF-DA fluorescent assay. Moreover, AAP exhibited stronger antioxidant activity when compared with the other plant-based polysaccharide-rich extracts including *Phyllophorus proteus* (EC_50_ > 1 mg/mL) [31] and *Ganoderma lucidum* (EC_50_ > 1 mg/mL) [35]. Importantly, AAP has been proven to possess antioxidant functions that assist in the reduction of skin inflammation.

Regarding the proliferation phase of the wound-healing process, new tissue formation is characterized by human fibroblast and keratinocyte proliferation, as well as their migration and invasion [4]. In contrast, impaired keratinocyte migration results in poor wound healing and the persistence of chronic wounds [23]. Our results demonstrated that human fibroblast and keratinocyte cells proliferation, migration, and invasion were induced by AAP in a dose-dependent manner. These results indicate wound-healing potential of AAP. Moreover, E-cadherin is the epithelial marker, which mediated cell–cell adhesion of epithelial cells. Previous studies reported that, the downregulation of E-cadherin promoted wound healing via increased epithelial cells invasion/migration [23,36]. Our results showed that AAP reduced the E-cadherin expressions in keratinocytes. Therefore, the downregulation of E-cadherin might be one of the wound-healing mechanisms of AAP. Apart from keratinocytes, during the proliferation state, fibroblasts also regulated the synthesis of the new ECM by secretion of collagen and other ECM proteins [2,4]. Our result showed that AAP could significantly stimulate collagen synthesis from human fibroblasts. Thus, the stimulation of collagen synthesis might be considered as another important wound-healing mechanism of AAP.

Based on the results of Sephacryl-S400 gel filtration and GC-MS studies, we can conclude that AAP is a homogeneous polysaccharide with a molecular weight of 158 kDa. The outcomes of our study strongly suggest that the wound-healing properties of AAP might be related to the rich content of polysaccharides in the extract. Our findings are in agreement with those of another study which reported on the molecular weight of *A. auricula* mushrooms [17]. Furthermore, the monosaccharides found in AAP (mannose, glucose, and galactose) were previously reported as either active compounds or a type of sugar backbone in the wound-healing agents based on the mushroom’s origins [37,38]. In terms of wound-healing properties, our data coincided with that of other reports which had used polysaccharide-rich extracts of either plants (*Bletilla striata* and *Astragalus membranaceus*) [39,40], seaweed (*Gracilaria lemaneiformis*) [24] or mushrooms (*Ganoderma lucidum, Agaricus blazei* and *Phellinus gilvus*) [41,42] to enhance keratinocyte and fibroblast cells proliferation or to accelerate the wound healing with other appropriate mechanisms [25]. Herein, this is the first report to demonstrate that polysaccharide-rich extract can stimulate the proliferation, migration, and invasion of skin keratinocytes and fibroblasts.

Our in vivo findings support the wound-healing potential of AAP as it could accelerate wound closure and improve the appearance of wound architecture without any cytotoxic effect in the mice skin wound-healing model. According to previous reports, skin that was either damaged or wounded was healed by the migration of the three main types of skin cells: keratinocytes, fibroblasts, and endothelial cells [43,44]. Without the migration of these cells, the main processes of wound healing cannot occur. This can lead to an unfinished wound-healing process and result in the delay of wound closure and the persistence of inflammation, which can then cause the formation of a chronic wound. Findings of previous in vivo studies on the wound-healing effects of medicinal mushrooms are in agreement with our results. Specifically, the polysaccharides obtained from *Gracilaria lemaneiformis* could induce wound healing in KM mice by accelerating the wound contraction rate, improving the epithelial layer thickness and collagen deposition, and decreasing inflammation [24]. Our data are the first report to display the in vivo wound-healing properties of polysaccharides obtained from the medicinal mushroom *A. auricula-judae*. Overall, our data reveal the benefits associated with the use of AAP as a potential candidate for wound-healing-accelerating agents due to its remarkable wound-healing properties identified in both in vitro and in vivo experiments.

## Data Availability

Not Applicable.
